# Dual targeting of ferroptosis and immune evasion: STAT3 inhibition as a one-two punch for breast cancer immunotherapy

**DOI:** 10.1515/jtim-2026-0074

**Published:** 2026-08-26

**Authors:** Jianbo Zhou, Cailu Song, Zhi Tian, Hailin Tang, Fu Peng

**Affiliations:** West China School of Pharmacy, Department of Thoracic Surgery and Institute of Thoracic Oncology, Frontiers Science Center for Disease-Related Molecular Network, West China Hospital, Sichuan University, Chengdu, Sichuan Province, China; State Key Laboratory of Oncology in South China, Guangdong Provincial Clinical Research Center for Cancer, Sun Yat-Sen University Cancer Center, Guangzhou, Guangdong Province, China; Department of Molecular Pharmacology & Physiology, Morsani College of Medicine, University of South Florida, Tampa, USA; West China School of Pharmacy, Key Laboratory of Drug-Targeting and Drug Delivery System of the Education Ministry, Sichuan Engineering Laboratory for Plant-Sourced Drug and Sichuan Research Center for Drug Precision Industrial Technology, Sichuan University, Chengdu, Sichuan Province, China

## Introduction

**Figure 1:**
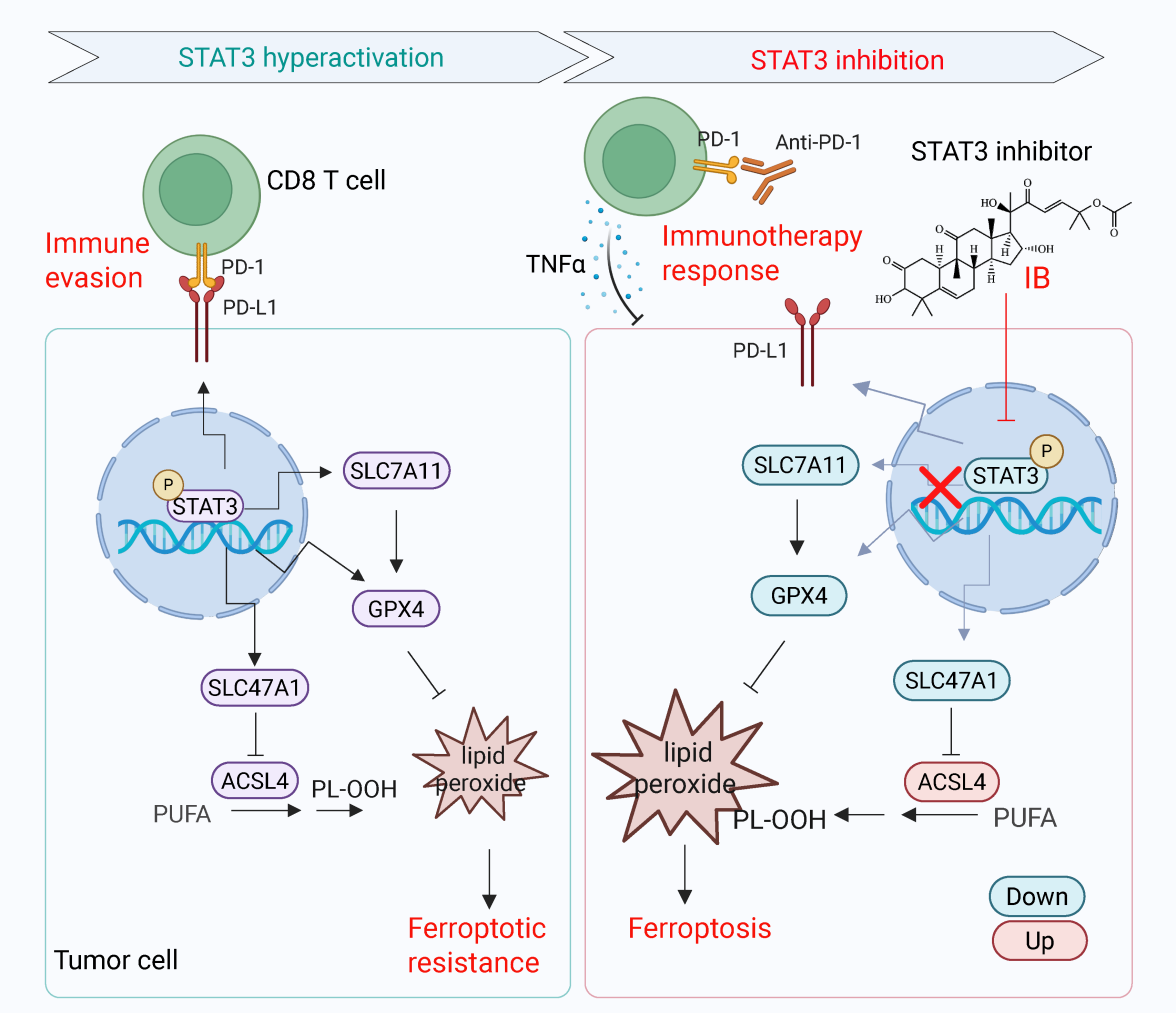
Mechanisms of STAT3 inhibition mediated by isocucurbitacin B to activate ferroptosis and mitigate immune evasion. STAT3: signal transducer and activator of transcription 3; ACSL4: Acyl-CoA synthetase long-chain family member 4; GPX4: glutathione peroxidase 4; IB: isocucurbitacin B; SLC7A11: solute carrier family 7 member 11; P: Phosphorylation; PD-1: programmed cell death protein 1; PD-L1: programmed death-ligand 1; PUFA: polyunsaturated fatty acids; PL-OOH: phospholipid hydroperoxides. Created in BioRender. Jianbo, Z. (2026) (https://BioRender.com/mh32ikj).

Signal Transducer and Activator of Transcription 3 (STAT3) is a well-characterized oncogenic transcription factor that has been extensively investigated. It is aberrantly activated across numerous cancer types and correlates with unfavorable clinical prognosis, driving tumor progression and immune evasion by upregulating programmed death-ligand 1 (PD-L1).^[1]^ Recent studies have further revealed that STAT3 also contributes to ferroptosis resistance through transactivating core anti-ferroptotic genes, including *SLC7A11*, *GPX4*, and *FTH1*.^[2]^ These lines of evidence collectively suggest that targeting STAT3 provides a two-pronged therapeutic strategy to simultaneously overcome ferroptosis resistance and cancer immune evasion.^[3]^ Recently, Zhou *et al*.^[4]^ reported in the *International Journal of Surgery* that STAT3 acts as a molecular hub connecting ferroptosis and immune evasion *via* directly transactivating *SLC47A1* and *PD-L1*, respectively, in their article entitled “Isocucurbitacin B targets STAT3 to induce ferroptosis and promote anti-PD-1 immunotherapy responses in breast cancer” (Figure 1). It also provides a potential molecular rationale for applying STAT3 inhibitors to potentiate breast cancer immunotherapy. While this study presents comprehensive and logically consistent work, several limitations remain that restrict its clinical translation and the generalizability of its conclusions.

## Pan-Cancer cell line screening reveals the anti-tumor potential of small molecules

The authors performed a limited panel of cell line screening and found that isocucurbitacin B (IB) exerted potent anti-proliferative effects on triple-negative breast cancer (TNBC) cell lines (MDA-MB-231 and BT-549)—the most aggressive and prognostically unfavorable breast cancer subtype—whereas minimal effects were observed in ER-positive breast cancer cells (MCF-7A). Accordingly, the authors selected TNBC cell lines for subsequent functional experiments. Notably, TNBC is stratified into four molecular subtypes: luminal androgen receptor (LAR), immunomodulatory (IM), basal-like immune-suppressed (BLIS), and mesenchymal-like (MES).^[5]^ Thus, these two cell lines, which belong to the MES subtype, are insufficient to fully recapitulate the clinical heterogeneity of TNBC. Further investigations are warranted to confirm the effects of IB across TNBC subtypes, which will facilitate the development of more precise and effective strategies for breast cancer precision therapy.

## Machine learning and label-free proteomics for small-molecule target identification

To identify direct protein targets of IB, the authors integrated three approaches, including proteomics-based DARTS-MS, transcriptome-based CMap analysis, and structural similarity screening against the Therapeutic Target Database (TTD). Among these, DARTS-MS directly detects increased protein abundance resulting from enhanced protein stability caused by drug-binding-induced protein stabilization, whereas the other two methods provide relatively indirect evidence. Taking the intersection of these three datasets may lead to the loss of bona fide proteins with strong-interactions. STAT3 was only one of 11 potential candidate targets and was not highly ranked in the DARTS-MS output, suggesting that STAT3 is unlikely to represent the primary or highest-affinity direct binding target of IB. Although the authors validated the physical interaction between IB and STAT3 using MST and CETSA-WB, further functional rescue experiments are still required. Ideally, IB-induced phenotypes should be largely abolished in STAT3-knockout or STAT3-knockdown cells to firmly establish that IB acts by targeting STAT3. In addition, integrating additional computational platforms, including Swiss Target Prediction, SEA, PPB2, and ChEMBL33, could help construct a more comprehensive landscape of candidate targets.^[6]^ Activity-based protein profiling (ABPP) can be utilized for candidate target identification and combined with DARTS-MS to improve the reliability of target screening.

## A novel ferroptosis-regulatory axis: STAT3/SLC47A1

A previous study has identified SLC47A1 as a ferroptosis suppressor, which mitigates RSL3- or erastin-induced ferroptosis by repressing ACSL4-mediated accumulation of polyunsaturated fatty acid–cholesterol esters.^[7]^ By integrating transcriptomic and ChIP-seq data, Zhou *et al*.^[4]^ first discovered that the transcriptional regulation of SLC47A1 by STAT3, advancing our understanding of STAT3-dependent ferroptotic regulation beyond the canonical STAT3/SLC7A11/GPX4 pathway. Furthermore, the authors confirmed, using two GEO datasets, that the STAT3/SLC47A1 axis is associated with poorer prognosis in breast cancer patients. Nevertheless, targeted validation in TNBC cohorts is warranted to strengthen these findings.

## Conclusion

In summary, STAT3 serves as a critical hub linking ferroptosis and tumor immunity *via* the transcriptional regulation of SLC47A1 and PD-L1, thereby uncovering a novel potential molecular mechanism that may improve immunotherapy responses in breast cancer. As supported by Zhou *et al*.^[4]^ STAT3 inhibitors (*e.g*., isocucurbitacin B) appear to exert a two-pronged effect by concurrently overcoming ferroptosis resistance and immune evasion.

STAT3 is hyperactivated in a wide range of solid malignancies, including breast cancer, lung cancer and ovarian cancer. Its aberrant activation is closely correlated with malignant progression, therapeutic resistance and poor prognosis, supporting its promise as a pivotal therapeutic target. The efficacy of STAT3 inhibition has been validated in preclinical cancer models, with representative small molecules including alicanthone, loliolide, and quercetin.^[8]^ Ongoing clinical studies are investigating the synergistic effects of combining STAT3 inhibition with immunotherapy (PD-1/PD-L1 inhibitors).^[9]^ Despite nearly three decades of efforts in developing STAT3-targeted anticancer drugs, no STAT3 inhibitor has been approved by the FDA for clinical cancer treatment to date.^[10]^ The discovery of novel STAT3 inhibitory scaffolds derived from natural products, coupled with in-depth elucidation of STAT3-mediated molecular mechanisms, will substantially promote the clinical translation of STAT3-targeted therapies. This Perspective provides a novel mechanistic strategy into enhancing immunotherapy efficacy: STAT3 inhibition not only suppresses tumor immune evasion but also reverses ferroptosis resistance in an SLC47A1-dependent manner.

However, further structure–activity relationship studies are still required to fully characterize the antitumor efficacy and safety profile of isocucurbitacin B in breast cancer, as well as the broader translational potential of the cucurbitacin family. Moreover, the regulatory mechanism by which isocucurbitacin B modulates the tumor immune microenvironment remains poorly defined and warrants further exploration.
